# Web-Based Intervention Effects on Mild Cognitive Impairment Based on Apolipoprotein E Genotype: Quasi-Experimental Study

**DOI:** 10.2196/14617

**Published:** 2020-05-07

**Authors:** Anthoula C Tsolaki, Magda Tsolaki, Niki Pandria, Eftychia Lazarou, Olymbia Gkatzima, Vasiliki Zilidou, Maria Karagianni, Zafiroula Iakovidou-Kritsi, Vasilios K Kimiskidis, Panagiotis D Bamidis

**Affiliations:** 1 Medical Physics Laboratory School of Medicine Aristotle University of Thessaloniki Thessaloniki Greece; 2 Department of Neurology Agios Pavlos General Hospital Thessaloniki Greece; 3 1st Department of Neurology American Hellenic Educational Progressive Association Hospital Aristotle University of Thessaloniki Thessaloniki Greece; 4 Panhellenic Institute of Neurodegenerative Diseases Thessaloniki Greece; 5 Laboratory of Medical Biology-Genetics Department School of Medicine Aristotle University of Thessaloniki Thessaloniki Greece; 6 Laboratory of Clinical Neurophysiology American Hellenic Educational Progressive Association Hospital Aristotle University of Thessaloniki Thessaloniki Greece

**Keywords:** mild cognitive impairment, APOE ε4, computerized training, exergaming, Alzheimer disease

## Abstract

**Background:**

Apolipoprotein E (APOE) ε4 allele is a major genetic risk factor for Alzheimer disease and mild cognitive impairment (MCI). Computer-based training programs can improve cognitive performance in elderly populations. However, the effects of computer-based interventions on MCI APOE ε4 carriers have never been studied before.

**Objective:**

The effects of different web-based interventions and the APOE isoform-specific differences in training outcomes are investigated.

**Methods:**

Using a quasi-experimental study design, 202 participants with MCI aged 60 years and older took part in three different intervention programs (physical and cognitive [Long-Lasting Memories, or LLM], cognitive [Active Control, or AC], or physical intervention [Physical Training Control, or PTC]) via an innovative information and communication technologies exergaming platform. Participants in each interventional group were subdivided into APOE ε4 carriers and non–APOE ε4 carriers. All participants underwent an extensive neuropsychological evaluation before and after the training, blood tests, and brain imaging.

**Results:**

All interventions resulted in multiple statistically significant cognitive benefits after the intervention. Verbal learning (California Verbal Learning Test: immediate recall test score—LLM: *P*=.04; AC: *P*<.001), working memory (digit span forward and backward test scores—AC: *P*=.03; PTC: *P*=.02 and *P*=.006, respectively), and long-term memory (California Verbal Learning Test: delayed recall test score—LLM: *P*=.02; AC: *P*=.002; and PTC: *P*=.02) were improved. There was no statistically significant difference among the intervention effects. APOE ε4 presence moderates intervention effects as the LLM intervention improved only their task-switching processing speed (Trail Making Test, Part B: *P*=.03) and the PTC intervention improved only the working memory (digit span backward: *P*=.03). No significant performance alteration was noted for the APOE ε4+ cognitive AC training group.

**Conclusions:**

None of the applied interventions could be identified as the optimal one; it is suggested, however, that combined cognitive and physical training and physical training via exergaming may be more effective for the high-risk MCI ΑPOE ε4+ subgroup.

## Introduction

### Mild Cognitive Impairment

Mild cognitive impairment (MCI) can be defined as the condition between normal aging and dementia [1]. By the time older people meet the criteria for MCI, they have already exhibited measurable cognitive decline, and most of them have also accumulated the neuropathologic hallmarks of Alzheimer disease [2]. Heterogeneous etiology can cause MCI. Due to this heterogeneity, its progression is uncertain; patients may remain stable for years, a few could improve, and others could progress to dementia. Based on recent data, MCI presents a progression rate of 38.7% over 12 to 60 months. However, the progression rate across individual studies is quite variable, ranging from 6% to 39% per year [3].

Even though MCI is regarded as a preclinical stage of Alzheimer disease or other types of dementia, studies report neuronal loss of about 36.5% already at that stage as well as synaptic dysfunction [4]. MCI patients, however, seem to retain sufficient neuroplasticity to benefit from nonpharmacological interventions, which may, in turn, delay the progression to dementia [5]. Since there is currently no other treatment of dementia than the palliative one, research efforts are focused on possible ways that could delay disease onset, such as diet, cognitive, and physical training.

### Nonpharmaceutical Interventions

The recent growing interest in investigating interventions capable of ameliorating or delaying aging and neurodegenerative effects has resulted in designing various projects for cognitive or/and physical training of the elderly [6]. There is no consensus about the best combination of training. However, findings suggest that interventions targeting multiple domains may be more effective and even provide a long-term benefit for individuals at risk [7].

Cognitive training is based on the idea that the brain function is modifiable even in old age [6]. It is claimed that cognitive training may contribute to the delay or even prevention of cognitive decline in older adults, although this claim remains controversial [8]. Cognitive improvement after cognitive training is generally associated with both compensatory and restorative mechanisms [9-12].

Physical training seems to promote multiple gains in both physical and cognitive states. Hippocampal neurogenesis [13], decrease of β-amyloid deposition [14], oxidative stress reduction [15], brain perfusion increase, and upregulation of neurotrophic factors [16] are a few of the widely studied and reported effects. These effects were presented as improved mood state, improved cognitive function, reduced comorbidities, and decreased risk of falls [17,18].

Combined physical and cognitive training may facilitate the neuroplasticity potential and enhance an individual’s capacity to respond to new demands, resulting in mutual enhancement [19].

Recent advances in information and communication technologies (ICTs) and health informatics offer new and elderly-friendly training on web-based platforms [20]. These platforms may also serve people with limited access to an organized day care center, in their own home or an assisted living/nursing home facility, occasionally from a distance with remote surveillance by specialized personnel. Technology-assisted solutions for elderly physical training through gaming, termed exergaming, have been increasingly investigated [21,22]. Validation of the effectiveness of these approaches is currently a top research priority [23].

### Apolipoprotein E

The ε4 allele of the apolipoprotein E (APOE) gene (APOE ε4) is the major genetic risk factor for Alzheimer disease. APOE ε4 carriers not only have a higher risk but also an earlier onset of Alzheimer disease by 10 to 20 years [24] in a gene dose-dependent manner. APOE ε4 (ε4/ε4) homozygotes compared with persons homozygous for risk-neutral APOE ε3 (ε3/ε3) may have up to 15 times the increased the risk for developing Alzheimer disease while APOE ε4 heterozygotes (ε4/ε3 or ε4/ε2) only have a 4 times higher risk [25]. Numerous studies have attempted to elucidate the underlying mechanism for APOE ε4 influences on Alzheimer disease onset and progression. It has been difficult to determine whether the APOE ε4 represents a gain of toxic function, a loss of neuroprotective function, or both [26]. It is noteworthy that APOE ε4 is associated not only with Alzheimer disease but also with altered brain metabolism and structure in young cognitively normal adults [27].

The presence of APOE ε4 significantly influences the progression of healthy elderly to MCI and Alzheimer disease, and the progression risk peaks between ages 70 and 75 years [28]. Studies are suggesting that the effect of the ε4 allele on cognitive decline is stronger in this earlier clinical stage in comparison with later and more severe stages [26]. Moreover, the more sedentary the lifestyle of the elderly person is, the higher the impact of APOE ε4 on cerebral amyloid deposition. However, not all APOE ε4 carriers will develop Alzheimer disease, thereby suggesting the interactive effects of APOE genotype with other genetic or environmental factors [25].

Our long-term study aims to investigate the cognitive effects of different computer-based interventions depending on the APOE isoform. Also, the follow-up reevaluations at 6, 12, and 24 months intend to determine which training program, if any, can postpone further cognitive decline and dementia onset. In this paper, which describes the first part of the study, the pre-post training evaluation of the interventional groups is presented. We assessed and compared the efficacy of the different web-based interventions and subsequently assessed whether the APOE genotype may influence the outcome. It is expected that such a finding may be useful for the improvement of the currently existing and future designs of web-based, technology-assisted therapeutic interventions.

## Methods

### Study Design

We used a quasi-experimental study design [29] to explore the efficacy of different interventions on participants with MCI. Participants were allocated between 2009 and 2017 into three different interventional groups. Based on previous efficacy evidence of the Long-Lasting Memories (LLM) intervention, an integrated ICT platform combining cognitive exercises with physical activity [19,30], we did not use a passive group in the pre-post assessment due to ethical considerations. Thirteen participants, however, were evaluated in the same pre- and postintervention period without participating in the intervention for personal reasons. All other passive data were retrospectively collected from the database records of the Greek Association of Alzheimer’s Disease and Related Disorders, matched for demographic data and initial diagnosis. This passive group was used only for the long-term follow-up.

Randomization of the participants was not feasible mainly for practical issues, time, and financial limitations of the study. Allocation to groups was driven by nonsystematic practical and logistic reasons (national holiday time, number of successfully screened participants at a given time point, etc) but was not influenced by participant choice, motivation, or compliance. The type of intervention applied each time in each place (spiritual center, open care center) was determined before the initiation of the screening procedure, and it was an open call to the elderly whether they were cognitively intact or not. Interventions took place both in the east and west areas of Thessaloniki, minimizing geographic socioeconomic differences of participants. From all the participants who enrolled in the LLM project, we analyzed those with an initial diagnosis of MCI fulfilling the selection criteria.

The training lasted about 8 to 12 weeks, and participants completed at least 24 sessions of cognitive training and 16 sessions of physical training. Screening evaluations were conducted 1 to 2 weeks before interventions, while posttest evaluations occurred 1 to 2 weeks after the end of the training. Neuropsychologists performing the pre- and postintervention neuropsychological assessments were generally different from those who administered the program.

Participant performance was assessed and compared among the different interventional groups to investigate a potential superiority of an intervention. Moreover, the performance of each interventional group, in each test between the two time points (pre-post), was assessed separately, investigating specific cognitive domain improvement. A second analysis, based on genotype, was performed subsequently. Each interventional group was subdivided into APOE ε4 carriers and non–APOE ε4 carriers. A comparison of each APOE subgroup among the interventional groups was performed to assess potential differences in efficacy in different genotypes. The performance of each APOE subgroup in the two time points was assessed separately within the interventional group as well.

### Participants

**Figure 1:**
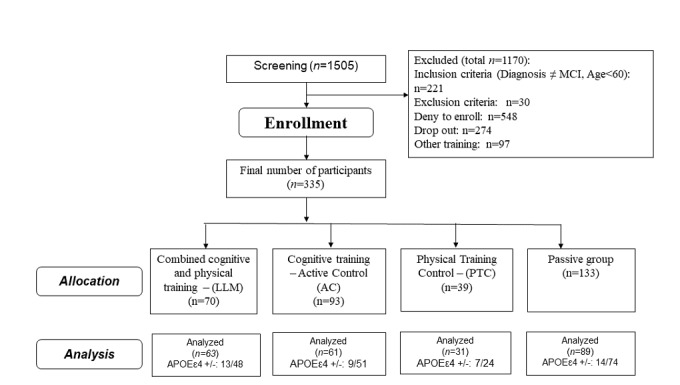
Study flowchart with the number of the participants screened and allocated in each interventional group. MCI: mild cognitive impairment; LLM: Long-Lasting Memories; APOE: apolipoprotein E.

A total of 215 MCI participants (intervention 202 [LLM 70; Active Control, or AC 93; Physical Training Control, or PTC 39] and passive 13; single and multiple domains) were recruited during a thorough screening procedure. The rest of the passive group data (n=120) was collected retrospectively as described in Study Design section (Figure 1). Males and females aged 60 years and older, fluent in Greek, were invited to participate. The call was made in church spiritual centers, open care centers for the elderly in east and west areas of Thessaloniki, and day care centers of the Greek Association of Alzheimer’s Disease and Related Disorders. Participants provided written informed consent and were compensated for their participation in the study. The study protocol was approved by the bioethics committee of the School of Medicine of the Aristotle University of Thessaloniki (protocol no 38/5.6.2013).

Exclusion criteria included any severe physical illness, current psychiatric or other neurological disorder (stroke, multiple sclerosis, Parkinson's disease, epilepsy, traumatic brain injury, etc), history of drug or alcohol abuse, and use of neuromodifying drugs (other than cholinesterase inhibitor).

All participants reported normal or corrected-to-normal vision and hearing. Before the training, all participants underwent an extensive neuropsychological evaluation performed using standardized Greek versions: Mini-Mental State Examination (MMSE) [31,32], Montreal Cognitive Assessment (MoCA) [33,34], and Trail Making Test, Part B (Trail B) [35,36] to examine task-switching, processing speed, and visuospatial ability; California Verbal Learning Test (CVLT) [37-39] and Rey Auditory Verbal Learning Test (RAVLT) in order to measure the ability of learning, long-term memory, and verbal episodic memory [40,41]; digit span forward and backward [42,43] to assess working memory; Instrumental Activities of Daily Living scale (IADL) [44,45], Functional Rating Scale of Symptoms of Dementia (FRSSD) [46], and Functional and Cognitive Assessment Test (FUCAS) [47] to assess daily functionality; Beck Depression Inventory (BDI) [48,49] and Geriatric Depression Scale (GDS) [50,51] to measure depression; and Beck Anxiety Inventory (BAI) [52,53] for anxiety evaluation. Participants were also subjected to blood tests and brain magnetic resonance imaging. A neurologist evaluated the neuropsychological, medical, and laboratory results. Τhe Cumulative Illness Rating Scale-Geriatric (CIRS) was calculated based on their medical history to assess participant comorbidity [54]. Diagnosis and categorization were based on clinical criteria [1,55] and were made by a dementia expert neurologist (MT). A detailed description of the procedure is described by Bamidis et al [19].

### Interventions

The first group attended the combined cognitive and physical training via the LLM project, using an integrated web service system through a universal interface, facilitated by touch screen systems [23,56]. Cognitive training was performed using a Greek-adapted version of the Brain Fitness software (later branded as BrainHQ, Posit Science Corporation) [57], and all physical exercises were implemented in the FitForAll (FFA) platform, an innovative ICT exergaming platform designed by Aristotle University of Thessaloniki [23]. FFA has four levels of difficulty and combines aerobic exercise; exercises of endurance, strength, and balance; and a cooling down process to recover normal cardiac rhythm.

The second group was the AC group, which was exposed to a homemade computerized cognitive training software suite (Video GRade, Lab of Medical Physics at the Aristotle University of Thessaloniki [58]), encompassing short documentary videos with themes from nature, art, history, and culture. At the end of each video, participants digitally performed a multiple-choice questionnaire about the documentary, following the same training dosage as the first group. The third group was the PTC group, which was exposed to computerized physical training using the FFA platform for the same number of sessions as the others. The fourth group was the passive control group, which did not follow any training program.

### Apolipoprotein E Genotyping

Blood samples used for genotyping were collected in EDTA-containing receptacles. DNA was extracted from peripheral blood using the QIAamp Blood DNA purification kit (Qiagen Inc). To determine the APOE genotype, part of the APOE gene (228 bp) containing both polymorphic sites (amino acid positions 112 and 158) was amplified by polymerase chain reaction analysis using the following primers: forward: 5″-GGCACGGCTGTCCAAGGAGCTGCA-3″ and reverse: 5″-GCCCCGGCCTGGTACACTGCCAG-3″, according to the method described by Koutroumani et al [59].

### Statistical Analysis

#### Multiple Imputation

In clinical and epidemiological research, the problem of missing data is almost unavoidable. In our study, missing data were handled using multiple imputation [60] tackling the missing data problem from three aspects: (a) the missing data proportion, (b) the mechanisms of incomplete data, and (c) the missing data patterns as suggested by Dong and Peng [61]. The missing data proportion was calculated, and missing data mechanisms were assessed using the Little multivariate test [62] and performing *t* tests of mean differences between the complete and missing data groups following the guidelines illustrated with a sample dataset from IBM SPSS Missing Values 20 [63]. The missing data pattern was explored using the command Analyze Patterns, which provides descriptive measures of the missing data patterns and could be useful as an exploratory step (see Multimedia Appendix 1 for methodology details). The analysis was performed using SPSS Statistics version 23 (IBM Corporation).

In our model, demographic data along with the scores of different neuropsychological tools and questionnaires were included following the guidelines incorporated in Dong and Peng [61]. Twenty imputations were chosen to remove noise from estimations, such as reducing sampling variability from the imputation procedure [64]. Following the completion of the multiple imputation, the 20 complete datasets were handled by standard statistical procedures after splitting the imputed dataset based on the imputation number. As many procedures in SPSS Statistics do not support pooling, we decided to report mainly pooled mean ranks that are naïve pooled. Even though we lose some of the descriptive power we could get from medians, we still gain valuable information about our groups in terms of our dependent variables. Moreover, we calculated the averaged median across the 20 complete datasets, as suggested by van Ginkel and Kroonenberg [65].

#### Demographics

During the baseline neuropsychological evaluation, a battery of tests was administered to participants including various neuropsychological tests and questionnaires assessing different cognitive aspects, performance in daily life activities, and the affective state of the participants. The scores collected by the tests and questionnaires used were tested for the normality assumption similarly to demographic data. As scores were not approximately normally distributed in all imputations, nonparametric analysis (Kruskal-Wallis test) was employed to explore differences between groups. When differences among groups reached statistical significance (*P*<.05), the Mann-Whitney *U* test was performed pairwise. Alpha inflation due to multiple comparisons was prevented using a Bonferroni correction.

Performance of participants was further discriminated depending on the presence of the APOE ε4 genotype. More precisely, scores on neuropsychological tests and questionnaires were also compared between APOE ε4 carriers versus non–APOE ε4 carriers. The normality assumption of test scores was explored, performing the described methodology between the two groups. As normality assumption was not met for both groups, a nonparametric analysis was performed (Mann-Whitney *U* test).

#### Neuropsychological Evaluation

##### Among-Group Analysis

Neuropsychological evaluations were administrated to the intervention groups (LLM, AC, and PTC) before and after the training completion. Scores of neuropsychological tests and questionnaires were analyzed using the group (LLM, AC, and PTC) as between factor and the time (pre- and posttraining) as within factor. The assumptions of mixed-model analysis of variance were not met in each cell of the design. Thus, an alternative analysis was followed using nonparametric procedures. Differences in scores at the two time points were computed for each test and questionnaire and then tested for normality. The Kruskal-Wallis H test was performed in differences (post-pre) of scores between groups. When differences among groups reached statistical significance (*P*<.05), the Mann-Whitney *U* test was performed pairwise. In this case, *P* values were corrected for multiple comparisons using a Bonferroni correction.

##### Within-Group Analysis

After grouping our dataset by the imputation number and group, Wilcoxon signed-rank tests were run to investigate possible differences of each group at different time points (pre- and posttraining) in M (M=20) complete datasets. Additionally, we examined a small sample of the passive group (n=13) regarding their performance in several tests such as the MMSE, MoCA, CVLT total, Trail B, IADL, and GDS at the two time points. Depending on normality assumption, different analyses were performed: either paired *t* test or Wilcoxon signed-rank test.

##### Among-Group Analysis Based on Genotype

Data were split not only by imputation number but also by genotype (carriers, non-ε4 carriers). Differences in scores at the two time points were computed for each test and questionnaire and then tested for normality. As normality assumption was not fulfilled for all imputations and groups, respectively, nonparametric analysis for between-group comparison was determined. Kruskal-Wallis H tests were run for the post-pre differences in scores having as grouping variable the group (LLM, AC, PTC). Results were split depending on the genotype. Significant differences among groups were explored running post hoc tests (pairwise comparisons using Mann Whitney *U* tests) and correcting for multiple comparisons.

##### Within-Group Analysis Based on Genotype

Data were split by imputation number, genotype (APOE ε4 carriers, non-ε4 carriers), and group. As violations of normality were observed, nonparametric analysis for within-comparison (pre, post) was performed.

## Results

### Demographics

A total of 335 participants in total were allocated to one of the four different groups (LLM, AC, PTC, passive). The baseline comparisons revealed significant differences between the four groups regarding demographics and cognitive performance. Therefore, a smaller sample of 244 subjects without differences in baseline evaluation scores was used for the pre-post comparison to evaluate the potential effect of the intervention without major confounding factors. Between and within-group comparisons at the first analysis stage were performed in LLM, AC, and PTC participants (see Table 1).

**Table 1:** Demographic characteristics of participants included in the study.

Groups		LLM^a^ n=70	AC^b^ n=93	PTC^c^ n=39	Passive n=133	Test results	
						χ^2^_3_	*P* value
Analyzed n=244		63	61	31	89		
**Age in years**						6.8	.08
	Median	69	69	67	68		
	IQR	7	9	8	14		
	Q1, Q3	66.0, 73.0	65.0, 74.0	63.0, 71.0	60.0, 74.0		
**Gender, n**						5.7	.13
	Male	51	46	28	63		
	Female	12	15	3	26		
**Education in years**						7.5	.06
	Median	6	8	6	8		
	IQR	7	6	1	6		
	Q1, Q3	6.0, 13.0	6.0, 12.0	6.0, 7.0	6.0, 12.0		
Comorbidity index (CIRS^d^), pooled mean ranks		121.39	105.11	129.53	132.76	6.4	.11
**APOE^e^ ε4/- (frequency % in total sample of 335 participants)**							
	APOE ε4 carriers	15 (21.4)	13 (14.0)	9 (23.1)	30 (22.6)		
	Non-APOE ε4 carriers	53 (75.7)	77 (82.8)	30 (76.9)	101 (75.9)		
**APOE ε4/- (frequency % in total sample of 244 participants)**							
	APOE ε4 carriers	13 (20.6)	9 (14.8)	7 (22.6)	14 (15.7)		
	Non-APOE ε4 carriers	48 (76.2)	51 (83.6)	24 (77.4)	74 (83.1)		

^a^LLM: Long-Lasting Memories.

^b^AC: Active Control.

^c^PTC: Physical Training Control.

^d^CIRS: Cumulative Illness Rating Scale–Geriatric.

^e^APOE: apolipoprotein E.

Discriminating the participants with regard to the presence of APOE ε4 genotype, we found that there were 43 APOE ε4 carriers, 197 non–APOE ε4 carriers, and in 4 cases there were missing data. Planned analysis of available data showed that the two groups did not differ in age (*U*=4022.00, *P*=.60), education years (*U*=4041.50, *P*=.62), and CIRS scores (*U*=3267.00, *P*=.69). Additionally, the two independent binomial proportions regarding the proportion of gender across groups were statistically significantly different (χ^2^_1_=3.9, *P*=.048; see Table 1).

In more detail, participants who were APOE ε4 carriers were barely older (age [pooled mean ranks] APOE ε4 carriers: 125.47; non–APOE ε4 carriers: 119.42) and more educated than noncarriers (education years [pooled mean ranks] APOE ε4 carriers: 125.01; non–APOE ε4 carriers: 119.52). Moreover, they had elevated CIRS scores relative to noncarriers (CIRS [pooled mean ranks] APOE ε4 carriers: 123.83; non–APOE ε4 carriers: 119.77).

### Neuropsychological Evaluation

#### Among-Group Analysis

Nonsignificant changes have been observed between groups in most of the neuropsychological tests comparing the scores’ differences at the two time points. Significant differences have been found only in the post-pre comparisons on the MMSE and GDS tests (Table 2). Based on the post hoc analysis, the LLM group seems to have a significantly lower performance on MMSE compared with AC participants and considerably more depressive symptoms relative to the PTC group.

**Table 2:** Presentation of among-group comparison results along with descriptive measures (pooled mean ranks of post-pre scores) for each group (Long-Lasting Memories, Active Control, and Physical Training Control).

Neuropsychological test		LLM^a^	AC^b^	PTC^c^	Test results	
		Pooled mean ranks	Pooled mean ranks	Pooled mean ranks	χ^2^_2_	*P* value
**Cognitive domain**						
	MMSE^d^	69.47	89.58	72.54	7.1	.04
	MoCA^e^	71.88	81.26	84.03	2.5	.34
	RAVLT1^f^	77.20	78.81	78.04	1.6	.52
	RAVLT total^g^	74.84	79.24	82.00	1.9	.48
	RAVLTD^h^	77.97	79.08	75.94	1.5	.53
	CVLT1^i^	69.37	84.09	83.57	5.0	.14
	CVLT total^j^	71.58	84.77	77.71	3.4	.26
	CVLTD^k^	71.33	79.23	89.14	4.7	.20
	Trail B^l^	74.95	81.23	77.84	1.0	.67
	Digit span forward	75.15	77.65	84.48	1.5	.52
	Digit span backward	72.73	78.77	87.19	2.6	.32
**Functionality**						
	FUCAS^m^	72.68	81.63	81.67	2.4	.39
	FRSSD^n^	74.47	83.83	73.70	3.9	.30
	IADL^o^	77.19	80.15	75.41	1.3	.61
**Affective domain**						
	GDS^p^	88.33	78.50	56.02	8.5	.005
	BAI^q^	76.33	80.74	75.99	0.1	.51
	BDI^r^	79.18	79.41	72.83	4.8	.49

^a^LLM: Long-Lasting Memories.

^b^AC: Active Control.

^c^PTC: Physical Training Control.

^d^MMSE: Mini-Mental State Examination.

^e^MoCA: Montreal Cognitive Assessment.

^f^RAVLT1: Rey Auditory Verbal Learning Test: immediate recall.

^g^RAVLT total: Rey Auditory Verbal Learning Test: sum up of 5 recall attempts.

^h^RAVLTD: Rey Auditory Verbal Learning Test: delayed recall.

^i^CVLT1: California Verbal Learning Test: immediate recall.

^j^CVLT total: California Verbal Learning Test: sum up of 5 recall attempts.

^k^CVLTD: California Verbal Learning Test: delayed recall.

^l^Trail B: Trail Making Test, Part B.

^m^FUCAS: Functional and Cognitive Assessment Test.

^n^FRSSD: Functional Rating Scale of Symptoms of Dementia.

^o^IADL: Instrumental Activities of Daily Living scale.

^p^GDS: Geriatric Depression Scale.

^q^BAI: Beck Anxiety Inventory.

^r^BDI: Beck Depression Inventory.

#### Within-Group Analysis

##### Long-Lasting Memories Group

LLM participants scored significantly higher in RAVLT1, RAVLT total, CVLT1, CVLT total, and CVLTD tests after their training compared with the baseline (Table 3). The within-group comparison did not reveal any significant differences either in functionality test scores of FUCAS, FRSSD, and IADL tests or emotion test scores of GDS, BAI, and BDI scores when comparing pre- and posttraining scores (Multimedia Appendix 2 Table A).

##### Active Control Group

The AC group showed significant improvement in their performance on the MMSE, RAVLT total, CVLT1, CVLT total, CVLTD, and digit span backward test when comparing the test scores before and after their training. They also showed significantly higher scores in the FUCAS test after training compared with the baseline evaluation, possibly indicating a decrease in their functionality in daily life activities (Table 3). Significant changes in the performance of the AC group at GDS, BAI, and BDI tests were not found when comparing scores at the two time points (Multimedia Appendix 2 Table B).

##### Physical Training Control Group

The PTC participants scored significantly higher in MoCA, RAVLT total, CVLT total, CVLTD, digit span forward, and digit span backward tests at the posttraining screening relative to the baseline. A significant decrease was also observed in scores on the GDS test at the posttraining neuropsychological screening relative to those of the baseline evaluation (Table 3; see Multimedia Appendix 3 Figure i for detailed results). Functionality scores of FUCAS, FRSSD, and IADL tests did not change significantly at the two time points (Multimedia Appendix 2 Table C).

The passive group (n=13), which was reassessed after the 12 weeks, did not reveal significant changes in their performance on the tests MMSE (t_12_=2.082; *P*=.059), MoCA (W=–0.319; *P*=.75), Trail B (t_11_=–0.656; *P*=.53), IADL (W=–0.577; *P*=.56), and GDS (W=–0.852; *P*=.39) at the two time points. However, a significant increase of 8.385 score units (95% CI 3.39 to 13.38, Cohen *d*=1.015 [38]) was found in CVLT total (t_12_=3.659; *P*=.003; CVLT baseline: 38.00; CVLT after 12 week: 46.38).

**Table 3:** Test scores with significant improvement for each interventional group when comparing their scores at the two time points.

Neuropsychological test		Pooled mean ranks: negative^a^	Pooled mean ranks: positive^b^	Averaged median before training	Averaged median after training	Test results	
						Wilcoxon signed-rank test	*P* value
**LLM^c^**							
	RAVLT1^d^	23.88	34.41	4.535	5.428	–3.2	.045
	RAVLT total^e^	25.45	32.65	37.121	41.85	–3.2	.04
	CVLT1^f^	20.35	29.62	4.795	5.311	–2.4	.04
	CVLT total^g^	25.22	31.18	41.369	46.206	–3.4	.002
	CVLTD^h^	19.67	30.73	8.559	9.239	–2.7	.02
**AC^i^**							
	MMSE^j^	22.56	24.96	27.000	28.000	–4.1	<.001
	RAVLT total	24.36	33.47	38.903	45.507	–3.8	.005
	CVLT1	20.36	30.79	4.584	6.650	–4.0	<.001
	CVLT total	16.94	32.25	39.941	47.742	–4.6	<.001
	CVLTD	23.74	30.98	8.259	9.908	–3.6	.002
	Digit span backward	21.30	26.96	4.004	4.488	–2.5	.03
**PTC^k^**							
	MoCA^l^	9.99	13.73	22.394	23.000	–2.0	.04
	RAVLT total	12.43	17.51	37.546	44.117	–2.7	.02
	CVLT total	11.56	16.41	41.187	48.359	–2.6	.02
	CVLTD	12.32	16.08	8.175	10.869	–3.3	.02
	Digit span forward	10.66	14.02	5.000	5.000	–2.3	.02
	Digit span backward	9.12	12.16	4.000	4.000	–2.8	.006
	GDS^m^	13.46	7.58	2.000	0	–3.5	.001

^a^Negative mean rank: test score post < test score pre.

^b^Positive mean rank: test score post > test score pre.

^c^LLM: Long-Lasting Memories.

^d^RAVLT1: Rey Auditory Verbal Learning Test: immediate recall.

^e^RAVLT total: Rey Auditory Verbal Learning Test: sum up of 5 recall attempts.

^f^CVLT1: California Verbal Learning Test: immediate recall.

^g^CVLT total: California Verbal Learning Test: sum up of 5 recall attempts.

^h^CVLTD: California Verbal Learning Test: delayed recall.

^i^AC: Active Control.

^j^MMSE: Mini-Mental State Examination.

^k^PTC: Physical Training Control.

^l^MoCA: Montreal Cognitive Assessment.

^m^GDS: Geriatric Depression Scale.

#### Among-Group Analysis Based on Genotype

##### Non-ε4 Carriers Among Groups

Non-ε4 carriers appear to significantly alter their performance neither in any cognitive tests nor in any test assessing their functionality in activities of daily living at the two time points depending on the group. Significant among-group differences were observed only in GDS tests. Planned post hoc tests revealed that LLM showed a considerably greater change in their geriatric depressive symptoms than the PTC group (Table 4 and Multimedia Appendix 3 Figure i).

##### Apolipoprotein E ε4 Carriers Among Groups

The ε4 carriers did not change their cognitive status significantly depending on the training given as evaluated by post-pre differences between groups. Furthermore, ε4 carriers seem to preserve their functionality and mood status as evaluated by respective tests at the two time points (Table 5 and Multimedia Appendix 3 Figure ii).

**Table 4:** Among-group comparison results in non-ε4 carriers. Descriptive measures (pooled mean ranks of post-pre scores) for each group (Long-Lasting Memories, Active Control, and Physical Training Control) are displayed.

Neuropsychological tests			LLM^a^		AC^b^		PTC^c^		Test results		
			Pooled mean ranks		Pooled mean ranks		Pooled mean ranks		χ^2^_2_		*P* value
**Cognitive domain**											
	MMSE^d^	57.11		70.00		54.77		4.6		.12	
	MoCA^e^	60.75		64.68		56.39		0.5		.80	
	RAVLT1^f^	61.13		62.48		62.73		1.7		.49	
	RAVLT total^g^	59.67		62.69		65.19		1.8		.51	
	RAVLTD^h^	62.93		61.79		60.60		1.7		.54	
	CVLT1^i^	53.86		67.73		66.11		5.3		.12	
	CVLT total^j^	56.74		67.69		60.43		3.2		.28	
	CVLTD^k^	57.78		61.58		71.33		3.6		.26	
	Trail B^l^	61.96		64.68		56.39		1.3		.58	
	Digit span forward	58.62		61.85		69.07		1.8		.44	
	Digit span backward	57.62		63.21		68.18		1.8		.45	
**Functionality**											
	FUCAS^m^	55.59		65.65		67.07		3.3		.27	
	FRSSD^n^	58.73		66.00		60.05		3.1		.35	
	IADL^o^	59.63		65.26		59.80		2.1		.48	
**Affective domain**											
	GDS^p^	69.64		61.65		47.46		6.6		.047	
	BAI^q^	60.18		64.09		56.13		1.9		.55	
	BDI^r^	64.13		62.76		56.13		2.3		.46	

^a^LLM: Long-Lasting Memories.

^b^AC: Active Control.

^c^PTC: Physical Training Control.

^d^MMSE: Mini-Mental State Examination.

^e^MoCA: Montreal Cognitive Assessment.

^f^RAVLT1: Rey Auditory Verbal Learning Test: immediate recall.

^g^RAVLT total: Rey Auditory Verbal Learning Test: sum up of 5 recall attempts.

^h^RAVLTD: Rey Auditory Verbal Learning Test: delayed recall.

^i^CVLT1: California Verbal Learning Test: immediate recall.

^j^CVLT total: California Verbal Learning Test: sum up of 5 recall attempts.

^k^CVLTD: California Verbal Learning Test: delayed recall.

^l^Trail B: Trail Making Test, Part B.

^m^FUCAS: Functional and Cognitive Assessment Test.

^n^FRSSD: Functional Rating Scale of Symptoms of Dementia.

^o^IADL: Instrumental Activities of Daily Living scale.

^p^GDS: Geriatric Depression Scale.

^q^BAI: Beck Anxiety Inventory.

^r^BDI: Beck Depression Inventory.

**Table 5:** Among-group comparison results in ε4 carriers. Descriptive measures (pooled mean ranks of post-pre scores) for each group (Long-Lasting Memories, Active Control, and Physical Training Control) are presented.

Neuropsychological tests		LLM^a^	AC^b^	PTC^c^	Test results	
		Pooled mean ranks	Pooled mean ranks	Pooled mean ranks	χ^2^_2_	*P* value
**Cognitive domain**						
	MMSE^d^	12.44	17.63	16.37	2.3	.31
	MoCA^e^	12.25	16.20	18.57	3.2	.25
	RAVLT1^f^	14.99	15.52	14.36	1.1	.66
	RAVLT total^g^	14.57	14.94	15.88	1.8	.49
	RAVLTD^h^	14.48	15.96	14.74	1.6	.57
	CVLT1^i^	14.09	14.67	17.12	1.6	.49
	CVLT total^j^	13.30	16.02	16.84	1.7	.50
	CVLTD^k^	13.37	15.69	17.15	1.9	.48
	Trail B^l^	12.16	15.52	19.61	4.1	.18
	Digit span forward	15.50	13.51	15.99	1.6	.61
	Digit span backward	14.42	14.68	16.49	0.8	.72
**Functionality**						
	FUCAS^m^	15.21	15.67	13.74	1.2	.59
	FRSSD^n^	15.25	15.99	13.25	2.0	.53
	IADL^o^	16.62	12.58	15.10	2.9	.45
**Affective domain**						
	GDS^p^	16.87	17.29	8.57	5.7	.07
	BAI^q^	15.07	15.86	13.77	1.8	.56
	BDI^r^	14.68	15.48	14.98	1.0	.64

^a^LLM: Long-Lasting Memories.

^b^AC: Active Control.

^c^PTC: Physical Training Control.

^d^MMSE: Mini-Mental State Examination.

^e^MoCA: Montreal Cognitive Assessment.

^f^RAVLT1: Rey Auditory Verbal Learning Test: immediate recall.

^g^RAVLT total: Rey Auditory Verbal Learning Test: sum up of 5 recall attempts.

^h^RAVLTD: Rey Auditory Verbal Learning Test: delayed recall.

^i^CVLT1: California Verbal Learning Test: immediate recall.

^j^CVLT total: California Verbal Learning Test: sum up of 5 recall attempts.

^k^CVLTD: California Verbal Learning Test: delayed recall.

^l^Trail B: Trail Making Test, Part B.

^m^FUCAS: Functional and Cognitive Assessment Test.

^n^FRSSD: Functional Rating Scale of Symptoms of Dementia.

^o^IADL: Instrumental Activities of Daily Living scale.

^p^GDS: Geriatric Depression Scale.

^q^BAI: Beck Anxiety Inventory.

^r^BDI: Beck Depression Inventory.

#### Within-Group Analysis Based on Genotype

##### Long-Lasting Memories Group

The ε4 carriers in the LLM group significantly improved their performance at Trail B while non-ε4 carriers considerably improved their performance on many cognitive tests such as the MMSE, MoCA, RAVLT1, RAVLT total, CVLT1, CVLT total, and CVLTK, comparing their test scores both before and after the training (Table 6 and Multimedia Appendix 3 Figure iii). Neither ε4 carriers nor non-ε4 carriers considerably altered their functional status in activities of daily living and depressive and anxiety symptomatology (Multimedia Appendix 2 Table D).

##### Active Control Group

AC ε4 carriers did not significantly change their scores on cognitive tests before and after training (Multimedia Appendix 2 Table E), while non-ε4 carriers showed a considerable improvement on the MMSE, MoCA, digit span backward, RAVLT total, an on different categories of the CVLT test. The ε4 carriers did not considerably change their functionality in activities of daily living as assessed by different tests at the two time points while the non-ε4 carriers significantly altered their scores on the FUCAS test, indicating a decrease in their functionality (Table 6 and Multimedia Appendix 3 Figure iv). Nonsignificant changes were observed in both ε4 carriers and non-ε4 carriers with regard to their depressive and anxiety symptomatology (Multimedia Appendix 2 Table E).

##### Physical Training Control

The ε4 carriers of PTC scored significantly better on digit span backward tests after training compared with the baseline, while non-ε4 carriers of the same group showed considerable improvement in a couple of tests such as RAVLT total, CVLT total, CVLTK, digit span forward, and digit span backward (Table 6 and Multimedia Appendix 3 Figure v).

PTC ε4 carriers did not show a significant change in their functionality in activities of daily living as assessed by the FUCAS, FRSSD, and IADL tests (Multimedia Appendix 2 Table F). However, non-ε4 carriers showed a marginally significant change only in FUCAS scores when comparing their scores at the two time points. Both ε4 carriers and non-ε4 carriers seemed to significantly improve their geriatric depressive scores after the training compared with the baseline screening (Table 6). Scores on BAI and BDI tests at the two time points did not change significantly in either group (Multimedia Appendix 2 Table F).

**Table 6:** Significant score changes in the performance of ε4 carriers and non-ε4 carriers of the Long-Lasting Memories, Active Control, and Physical Training Control groups.

Neuropsychological tests		Pooled mean ranks: negative^a^		Pooled mean ranks: positive^b^		Averaged median before training		Averaged median after training		Test results			
										Wilcoxon signed-rank test		*P* value	
**LLM^c^ ε4 carriers**													
	Trail B^d^		7.03		6.87		234.797		185.516		–2.2		.03
**LLM non-ε4 carriers**													
	MMSE^e^		14.30		20.36		27.000		28.000		–2.0		.047
	MoCA^f^		20.24		22.85		22.915		24.000		–2.2		.04
	RAVLT1^g^		17.33		26.15		4.523		5.463		–3.0		.049
	RAVLT total^h^		18.95		26.06		37.699		43.220		–3.0		.04
	CVLT1^i^		14.47		21.42		4.751		5.566		–2.6		.03
	CVLT total^j^		18.88		22.97		41.605		46.994		–3.4		.002
	CVLTD^k^		14.49		22.83		8.471		9.598		–2.7		.02
**Active non-ε4 carriers**													
	MMSE		19.58		21.34		26.948		28.000		–3.8		<.001
	MoCA		17.28		27.77		22.997		24.726		–2.4		.04
	RAVLT total		19.28		28.41		38.771		45.806		–3.6		.01
	CVLT1		14.54		26.21		4.507		6.832		–4.2		<.001
	CVLT total		12.45		26.75		39.180		48.408		–4.5		<.001
	CVLTD		19.06		25.64		8.312		10.002		–3.5		.002
	Digit span backward		17.41		22.16		4.000		4.362		–2.3		.046
	FUCAS^l^		17.66		23.69		43.798		44.271		–3.2		.004
**PTC^m^ ε4 carriers**													
	Digit span backward		2.50		3.75		4.000		5.000		–2.1		.03
	GDS^n^		3.50		0		2.000		0		–2.2		.03
**PTC non-ε4 carriers**													
	RAVLT total		9.37		13.62		37.509		44.118		–2.6		.03
	CVLT total		9.32		13.02		41.230		47.803		–2.4		.04
	CVLTD		9.41		13.16		7.871		10.822		–3.0		.03
	Digit span forward		9.02		10.96		4.405		5.000		–2.5		.01
	Digit span backward		6.52		9.96		4.000		4.000		–2.2		.03
	FUCAS		8.54		14.55		43.537		44.505		–2.3		.05
	GDS		10.68		5.28		2.000		0		–2.8		.006

^a^Negative mean rank: test score post < test score pre.

^b^Positive mean rank: test score post > test score pre.

^c^LLM: Long-Lasting Memories.

^d^Trail B: Trail Making Test, Part B.

^e^MMSE: Mini-Mental State Examination.

^f^MoCA: Montreal Cognitive Assessment.

^g^RAVLT1: Rey Auditory Verbal Learning Test: immediate recall.

^h^RAVLT total: Rey Auditory Verbal Learning Test: sum up of 5 recall attempts.

^i^CVLT1: California Verbal Learning Test: immediate recall.

^j^CVLT total: California Verbal Learning Test: sum up of 5 recall attempts.

^k^CVLTD: California Verbal Learning Test: delayed recall.

^l^FUCAS: Functional and Cognitive Assessment Test.

^m^PTC: Physical Training Control.

^n^GDS: Geriatric Depression Scale.

## Discussion

### Principal Findings

To our knowledge, this is the first quasi-experimental study to investigate in elders with MCI the impact of a combination of computerized physical and cognitive training not only in terms of cognitive decline in general but also based on the different APOE isoforms. Our results indicate that MCI APOE ε4 carriers respond differently and less prominently in web-based interventions.

Since there are no previous data about nonpharmacological computerized interventional outcomes on APOE ε4 carriers, we cannot possibly make direct comparisons with past research, but we do underline the importance of these new findings and the likely key role of future investigations with longitudinal randomized trials.

There are, however, recent data on computerized training interventions in MCI subjects that present results in line with our findings, suggesting improvement of learning ability (RAVLT total, CVLT total) and short-term memory (RAVLT1, CVLT1, digit span), verbal memory (RAVLT, CVLT), task-switching, processing speed and visuospatial memory (Trail B), episodic memory (RAVLTD and CVLTD delayed recall) and attention (MMSE, MoCA), and a positive effect on depressive symptoms (GDS) as well (for a review see Klimova et al [66]). Although these outcomes appear promising, researchers underline the limitations of these studies because of their duration, small sample sizes, and methodological differences. A recent meta-analysis on classic cognitive interventions targeting multiple domains in MCI subjects presented cognitive benefits in working memory, attention, and verbal memory in a larger study series [5]. The above outcomes, which are consistent with ours, enhance the strength of the thesis that training in the MCI population is a promising tool against neurodegeneration.

The combination of physical and cognitive training has also been studied previously in this high-risk population, underlining not only the cognitive benefits [67,68] but also the improvement of biological parameters such as brain-derived neurotrophic factor levels, grey matter volume [69], and increased para-hippocampal cerebral blood flow [67].

Recent data on exergaming, using the latest technology of the virtual reality with low and high cognitive engagement, also demonstrated improvement in verbal memory, a cognitive parameter that showed improvement in all of our interventional groups [69].

In our study, the analysis among the groups did not reveal statistically significant differences in the effect of the three different interventions other than the MMSE (LLM<AC) and the GDS (LLM>PTC), so we could not possibly identify the best intervention. Assessment of activities of daily living revealed no differences, while changes in the affective domain underlined the positive effect of physical exercise on stress and depression.

Within each interventional group, statistically significant differences were more obvious. The LLM group showed significant improvement in episodic memory, learning ability, and long-term memory, while no significant changes existed in functionality and the affective domain. The AC group showed a considerable improvement in global cognition status as assessed by MMSE, episodic memory, learning ability, long-term memory, and working memory. Finally, the PTC group showed significant improvement in global cognitive status as assessed by MoCA and in working memory, learning ability, and long-term memory. The PTC group also had statistically diminished depressive symptoms, which is also repeatedly shown in literature to be a beneficial effect of physical exercise in mood disorders [70].

The small sample of the passive group reassessed at 12 weeks presented no change in most of the parameters under investigation. They only showed an improvement in learning ability. This result may be explained by the short interval between the pre-post evaluation and familiarization with the evaluation process.

When participants were divided by genotype into two subgroups of APOE ε4 carriers and non-ε4 carriers, comparisons among the groups showed statistically significant differences only for the non-ε4 carriers’ depressive symptoms assessed by GDS between the LLM and PTC groups, with a less depressive burden for the PTC group. The ε4 carriers did not differentiate for any parameter between the groups.

Within-group analysis, however, revealed statistically significant improvements in the LLM group in working memory and visuospatial ability as measured by the Trail B test for the ε4 carriers, while the non-ε4 carriers showed considerable improvements in multiple cognitive domains. No changes have been noticed for either of the subgroups regarding functionality and emotional burden (Multimedia Appendix 3 Figure iii).

The AC ε4 carrier group showed no significant improvement in cognitive, functional, or emotional status. The non-ε4 carrier subgroup revealed multiple domain improvement once again but also a mild worsening of their functionality assessed by FUCAS. This functional decline drives us to the assumption that physical activity helps the maintenance of complex functional tasks in comparison with cognitive training alone (Multimedia Appendix 3 Figure iv).

Finally, the PTC ε4 carrier group showed the only improvement in working memory while non-ε4 carriers had significant improvement in multiple domains (RAVLT1, CVLT total, CVLTD, digit span). The non-ε4 carriers in this group also had slightly worse functionality assessed by the FUCAS. Both subgroups, however, showed a statistical improvement in depressive symptoms (Multimedia Appendix 3 Figure v).

APOE isoforms have been used as potential predictor markers for examining cognitive intervention effects in the current literature. Peter et al [71] suggest that the presence of APOE ε4 was not a significant predictor of any change in the cognitive variables, while another study underlines the potential beneficial effect of its absence [72]. The above findings are in line with ours, as we found stability in cognitive performance in the case of ε4 carriers and a significant improvement in the case of non-ε4 carriers.

The presence of APOE ε4 has also been related to biological and cognitive outcomes in physical exercise. Different brain metabolic responses to exercise related to the APOE isoforms have been reported [73], although their significance remains to be elucidated. Hence, Makino et al [74] suggest potential memory function benefits of physical exercise for ε4 carriers among older adults, also consistent with our findings, showing improvement in working memory in both groups that used physical training.

In our study, the ΑPOE ε4 presence was related to a resistance of cognitive improvement, while the non-ε4 carriers showed multiple cognitive benefits. Although nonresponders, the ε4 carriers seemed to improve at least one of the test scores under investigation with combined cognitive and physical training or physical training via exergaming but not with cognitive training only. That may be explained by the beneficial effects of physical exercise activity on systematic and neurological biological parameters [67,69,73-76]. The question that stems from the above findings is whether this is an interventional failure or the success of the disease progression postponement. The answer to the above question will be presented in a future paper on this study.

### Limitations

Our study has certain limitations. Randomization and blinding of test administrators and participants was not feasible due to practical issues. However, the lack of randomization is unlikely to bias effects as demographic characteristics and baseline performance are comparable. In our sample, 17.6% (43/244) were ΑPOE ε4 carriers (at least one ε4 allele), which is lower than the expected Greek population frequency of 25.5% [77]. That is due to the small sample size (n=244) regarding genetic studies, although other cognitive intervention studies recruited even fewer participants (n<100 [72,73]).

The short time to reevaluation may influence the described effect on neuropsychological test scores. However, the short-term interval of 12 weeks for intervention is commonly used in the design of similar studies. The missing values issue is a common problem in clinical research. It was handled in the most effective and statistically approved way as described in the literature, considering all the parameters and running all the necessary tests to avoid statistical analyses bias. Regarding future review and meta-analysis, we should declare that 35 participants in the LLM group were part of the interventional group of a previous study [19]. Future research in the field should consider these difficulties and may overcome them by using larger samples and long-term follow-up.

### Conclusions

Exergaming is an effective intervention method for patients with MCI. None of the applied computer-based interventions could be identified as the best. Nevertheless, it seems that combined cognitive and physical training and physical training via exergaming tend to be more effective for the high-risk MCI ΑPOE ε4+ subgroup.

## Appendix

Multimedia Appendix 1 Statistical analysis.

Multimedia Appendix 2 Supplementary tables.

Multimedia Appendix 3 Detailed results.

## Abbreviations

AC: Active Control
APOE: apolipoprotein E
APOE ε4: ε4 allele of the apolipoprotein gene
BAI: Beck Anxiety Inventory
BDI: Beck Depression Inventory
CIRS: Cumulative Illness Rating Scale–Geriatric
CVLT: California Verbal Learning Test
FFA: FitForAll
FRSSD: Functional Rating Scale of Symptoms of Dementia
FUCAS: Functional and Cognitive Assessment Test
GDS: Geriatric Depression Scale
IADL: Instrumental Activities of Daily Living scale
ICT: information and communication technologies
LLM: Long-Lasting Memories
MCI: mild cognitive impairment
MMSE: Mini-Mental State Examination
MoCA: Montreal Cognitive Assessment
PTC: Physical Training Control
RAVLT: Rey Auditory Verbal Learning Test
Trail B: Trail Making Test, Part B
