# Supplementary material for: Web-Based Intervention Effects on Mild Cognitive Impairment Based on Apolipoprotein E Genotype: Quasi-Experimental Study
Source: J Med Internet Res. 2020 May 7;22(5):e14617. doi: 10.2196/14617 (PMC7243129; doi:10.2196/14617)
Supplement: Multimedia Appendix 1 [file jmir_v22i5e14617_app1.docx]

**Missing values Treatment Methodology**

In clinical and epidemiological research, the problem of missing date is almost unavoidable. However, different missing data treatments could have serious impact on validity of research results (Sterne et al., 2009a; Wood et al., 2004). More precisely, missing data could lead to biased parameter estimation weakening the generalizability of the outcomes (Donald, 1987; Schafer, 1997). Additionally, analyzing data based on complete cases results in information loss along with decreasing power and precision as well as increasing standard errors(Peng et al., 2006; Sterne et al., 2009b). Since the statistical procedures have been designed for complete datasets, failing to preprocess the data properly could lead to unsuitable data for further statistical analyses(Dong and Peng, 2013a).

Among studies reporting missing data, the majority of them (97%) have used either listwise deletion (LD) or pairwise deletion (PD) when coping with incomplete data. Both methods are ad hoc techniques and well known for inefficient and/or biased estimates in most cases (Donald, 1987; Schafer, 1997). The APA Task Force on Statistical Inference advised against their usage (Wilkinson, 1999). Moreover, other methods for replacing missing values with imputed values from the observed data seem to statistically invalid because they introduce serious bias. In general, single imputation leads to decreased standard errors and fails to account for the uncertainty of missing values (Sterne et al., 2009b). Principled methods such as the multiple imputation (MI), the expectation-maximization (EM) method and the full information maximum likelihood (FIML), take account for the conditions under incomplete data occurred combining information of the observed data with statistical assumptions and they further estimate the population parameters and missing data mechanisms in a statistical manner (Dong and Peng, 2013b). We focused on multiple imputation method as it is a popular alternative among the others. MI handles the missing data problem in three steps (1) imputing incomplete data *m* times and generating *m* complete datasets, (2) analyzing *m* complete datasets following standard statistical procedures and (3) combining *m* results into one pooled result.

In our dataset, missing data were handled using multiple imputation tackling the missing data problem from three aspects: (a) the missing data proportion, (b) the mechanisms of incomplete data and (c) the missing data patterns as suggested by Dong and Peng (2013). The software used was the IBM SPSS Statistics, Version 23. The missing data proportion was calculated. Since an established cutoff regarding the acceptable percentage of missing data does not exist and the impact of missing data mechanisms and patterns is greater than their proportion, we continued the multiple imputation application even if we had a great number of incomplete data (Tabachnick and Fidell, 2012). The missing data mechanisms were assessed using Little’s multivariate test (Little and Schenker, 1995) and performing *t-tests* of mean differences between the complete and missing data groups following the guidelines illustrated with a sample dataset at IBM SPSS Missing Values 20^[[1]](#footnote-1)^. Moreover, the missing data pattern was determined using the command Analyze Patterns. It provides descriptive measures of the missing data patterns and it could be useful as an exploratory step.

Multiple imputation has the potential to ameliorate medical research validity by modeling the distribution of each missing value’s variable depending on observed data. The validity of the analysis should be based on several assumptions such as multivariate normality assumption, the imputations’ number and the convergence of the used model. However, MI based on multivariate normal model can provide reliable and valid results even when the assumption of normality is violated (Demirtas et al., 2008; Schafer, 1997, 1999). Additionally, when missing variables are categorical or non-normal the fully conditional specification (also called chained equations) is used as the preferred imputation model. Thus, multiple imputation provide the potential to handle variables that do not meet the normality assumption, as in our case, without the need of transforming the original data as proposed by other studies (Sterne et al., 2009b). This assumption is robust when missing rate is low and the sample size is large. However, the adequate sample size and the missing rate have not been specified in the literature (Schafer, 1997). Regarding the optimal number of imputations, methodology experts have not agreed. Although, Schafer and Olsen (1998) suggested that three to five imputations could be sufficient to have reliable results, Schafer and Graham (2002) proposed that twenty imputations are needed to remove noises from estimations such as reducing sampling variability from the imputation procedure (Sterne et al., 2009b). In our model, demographic data along with the scores of different neuropsychological tools and questionnaires were included following the guidelines incorporated in (Dong and Peng, 2013a). Finally, the convergence of the fully conditional specification method was checked by plotting the means and the standard deviations by iteration and imputation (for each dependent variable for which values were imputed as including too many variables could lead to non-convergence) (Dong and Peng, 2013a).

Before launching the imputation process, we set a random seed in order to have the opportunity to replicate the analysis exactly using the option of Random Number Generators as suggested by the guidelines illustrated in the IBM SPSS Missing Values 20. Following the completion of the MI, the twenty complete datasets were handled by the standard statistical procedures after splitting the imputed dataset based on the imputation number. Since many procedures in SPSS did not support pooling of results from analysis of multiple imputed datasets, pooled parameters were calculated as the mean of *m* estimates as suggested by (van Ginkel and Kroonenberg, 2014)

Demirtas, H., Freels, S. A., and Yucel, R. M. (2008). Plausibility of multivariate normality assumption when multiply imputing non-Gaussian continuous outcomes: a simulation assessment. *J. Stat. Comput. Simul.* 78, 69–84. doi:10.1080/10629360600903866.

Donald, B. R. (1987). *Multiple Imputation for Nonresponse in Surveys*. New York: John Wiley & Sons, Inc.

Dong, Y., and Peng, C.-Y. J. (2013a). Principled missing data methods for researchers. *Springerplus* 2, 222. doi:10.1186/2193-1801-2-222.

Dong, Y., and Peng, C.-Y. J. (2013b). Principled missing data methods for researchers. *Springerplus* 2, 222. doi:10.1186/2193-1801-2-222.

Little, R., and Schenker (1995). “Missing Data,” in *Handbook of Statistical Modeling for the Social and Behavioral Sciences*, ed. S. M. Arminger G, Clogg CC (New York: Plenum Press), 39–75.

Peng, C.-Y. J., Harwell, M., Liou, S.-M., Ehman, L. H., and others (2006). Advances in missing data methods and implications for educational research. *Real data Anal.* 10, 31–78. doi:10.1186/1476-072X-10-67.

Schafer, J. (1997). *Analysis of incomplete multivariate data*. London: Chapman & Hall/CRC.

Schafer, J. L. (1999). Multiple imputation: a primer. *Stat. Methods Med. Res.* 8, 3–15. doi:10.1177/096228029900800102.

Schafer, J. L., and Graham, J. W. (2002). Missing data: Our view of the state of the art. *Psychol. Methods* 7, 147–177. doi:10.1037/1082-989X.7.2.147.

Schafer, J. L., and Olsen, M. K. (1998). Multiple Imputation for Multivariate Missing-Data Problems: A Data Analyst’s Perspective. *Multivariate Behav. Res.* 33, 545–571. doi:10.1207/s15327906mbr3304_5.

Sterne, J. A. C., White, I. R., Carlin, J. B., Spratt, M., Royston, P., Kenward, M. G., et al. (2009a). Multiple imputation for missing data in epidemiological and clinical research: potential and pitfalls. *BMJ* 338, b2393–b2393. doi:10.1136/bmj.b2393.

Sterne, J. A. C., White, I. R., Carlin, J. B., Spratt, M., Royston, P., Kenward, M. G., et al. (2009b). Multiple imputation for missing data in epidemiological and clinical research: potential and pitfalls. *BMJ* 338, b2393–b2393. doi:10.1136/bmj.b2393.

Tabachnick, B., and Fidell, L. (2012). *Using multivariate statistics*. Allyn & Bacon, Needham Heights, MA.

van Ginkel, J. R., and Kroonenberg, P. M. (2014). Analysis of Variance of Multiply Imputed Data. *Multivariate Behav. Res.* 49, 78–91. doi:10.1080/00273171.2013.855890.

Wilkinson, L. (1999). Statistical methods in psychology journals: Guidelines and explanations. *Am. Psychol.* 54, 594–604. doi:10.1037/0003-066X.54.8.594.

Wood, A. M., White, I. R., and Thompson, S. G. (2004). Are missing outcome data adequately handled? A review of published randomized controlled trials in major medical journals. *Clin. Trials* 1, 368–376. doi:10.1191/1740774504cn032oa.

1. <ftp://public.dhe.ibm.com/software/analytics/spss/documentation/statistics/20.0/en/client/Manuals/IBM_SPSS_Missing_Values.pdf> [↑](#footnote-ref-1)
