# Supplementary material for: Web-Based Intervention Effects on Mild Cognitive Impairment Based on Apolipoprotein E Genotype: Quasi-Experimental Study
Source: J Med Internet Res. 2020 May 7;22(5):e14617. doi: 10.2196/14617 (PMC7243129; doi:10.2196/14617)
Supplement: Multimedia Appendix 2 [file jmir_v22i5e14617_app2.docx]

|  | Table 1: Demographic characteristics of the participants included in the study. | | | | | | |
| --- | --- | --- | --- | --- | --- | --- | --- |
| **GROUPS** | | | **LLM** | **ACTIVE** | **PTC** | **PASSIVE** | **Test Results** |
| **Total** N= 335 | | | N=70 | N=93 | N=39 | N=133 |  |
| **Analyzed** n=244 | | | n=63 | n=61 | n=31 | n=89 |  |
| **AGE** (years) | | |  |  |  |  |  |
|  | | Median | 69.0 | 69.0 | 67.0 | 68.0 | *χ^2^(3)=6.772, P= .08* |
|  | | Interquartile range | 7.0 | 9.0 | 8.0 | 14.0 |  |
|  | | [Q_1_, Q_3_] | [66.0, 73.0] | [65.0, 74.0] | [63.0, 71.0] | [60.0, 74.0] |  |
| **GENDER** (Female/Male) | | | 51/12 | 46/15 | 28/3 | 63/26 | *χ^2^(3)=5.698, P= .13* |
| **EDUCATION** (years) | | |  |  |  |  |  |
|  | | Median | 6.0 | 8.0 | 6.0 | 8.0 | *χ^2^(3)=7.456, P= .06* |
|  | | Interquartile range | 7.0 | 6.0 | 1.0 | 6.0 |  |
|  | | [Q1, Q3] | [6.0, 13.0] | [6.0, 12.0] | [6.0, 7.0] | [6.0, 12.0] |  |
| **Co morbidity Index** (CIRS)  (Pooled Mean Ranks) | | | 121.39 | 105.11 | 129.53 | 132.76 | *χ^2^(3)=* *6.444, P=* *.11* |
| **APOE ε4/-** (Frequency % in total sample of 335 participants) | | | 15/53  21.4%/75.7%  Missing: 2  (2.9%) | 13/77  14.0%/82.8%  Missing: 3  (3.2%) | 9/30  23.1%/76.9%  Missing: 0  (0.0%) | 30/101  22.6%/75.9%  Missing: 2  (1.5%) |  |
| **APOE ε4/-** (Frequency % in total sample of 244 participants) | | | 13/48  20.6%/76.2%  Missing: 2  (3.2%) | 9/51  14.8%/83.6%  Missing: 1  (1.6%) | 7/24  22.6%/77.4%  Missing: 0  (0.0%) | 14/74  15.7%/83.1%  Missing: 1  (1.1%) |  |

**Table 2**: Presentation of among-groups comparisons results along with descriptive measures (pooled mean ranks of post-pre scores) for each group (LLM, Active, PTC).

| Neuropsychological tests | LLM | Active | PTC | Test results |
| --- | --- | --- | --- | --- |
| ***Cognitive Domain*** | | | | |
|  | *Pooled Mean Ranks* | *Pooled Mean Ranks* | *Pooled Mean Ranks* |  |
| MMSE | *69.47* | *89.58* | *72.54* | *χ^2^(2)=* *7.059, P= .036^a^* |
| MoCA | *71.88* | *81.26* | *84.03* | *χ^2^(2)=* *2.462, P= .34* |
| RAVLT1 | *77.20* | *78.81* | *78.04* | *χ^2^(2)=* *1.631, P=* *.52* |
| RAVLT total | *74.84* | *79.24* | *82.00* | *χ^2^(2)=* *1.872, P= .48* |
| RAVLTD | *77.97* | *79.08* | *75.94* | *χ^2^(2)=* *1.519, P= .53* |
| CVLT1 | *69.37* | *84.09* | *83.57* | *χ^2^(2)=* *5.042, P= .14* |
| CVLT total | *71.58* | *84.77* | *77.71* | *χ^2^(2)=* *3.443, P= .26* |
| CVLTD | *71.33* | *79.23* | *89.14* | *χ^2^(2)=* *4.654, P= .20* |
| Trail B | *74.95* | *81.23* | *77.84* | *χ^2^(2)=* *0.963, P= .67* |
| Digit Span Forward | *75.15* | *77.65* | *84.48* | *χ^2^(2)=* *1.474, P= .52* |
| Digit Span Backward | *72.73* | *78.77* | *87.19* | *χ^2^(2)=* *2.569, P= .32* |
| ***Functionality*** | | | | |
| FUCAS | *72.68* | *81.63* | *81.67* | *χ^2^(2)=* *2.392, P= .39* |
| FRSSD | *74.47* | *83.83* | *73.70* | *χ^2^(2)=* *3.873, P= .30* |
| IADL | *77.19* | *80.15* | *75.41* | *χ^2^(2)=* *1.283, P= .61* |
| ***Affective Domain*** | | | | |
| GDS | *88.33* | *78.50* | *56.02* | *χ^2^(2)=* *8.481, P= .005^a^* |
| BAI | *76.33* | *80.74* | *75.99* | *χ^2^(2)=* *0.148, P= .51* |
| BDI | *79.18* | *79.41* | *72.83* | *χ^2^(2)=* *4.762, P= .49* |

*^a^ statistically significant value with P< .05*

Table 3: Test scores with significant improvement for each interventional group, when comparing their scores at the two-time points.

| Neuropsychological tests | LLM | | | | Test results |
| --- | --- | --- | --- | --- | --- |
|  | *Pooled Mean Ranks*  *Negative* | *Pooled Mean Ranks*  *Positive* | *Averaged Median*  *Before Training* | *Averaged Median*  *After Training* |  |
| RAVLT1 | *23.88* | *34.41* | *4.535* | *5.428* | *W=-3.146, P= .045* |
| RAVLT total | *25.45* | *32.65* | *37.121* | *41.85* | *W= -3.198, P= .035* |
| CVLT1 | *20.35* | *29.62* | *4.795* | *5.311* | *W= -2.424, P= .041* |
| CVLT total | *25.22* | *31.18* | *41.369* | *46.206* | *W= -3.383, P= .002* |
| CVLTD | *19.67* | *30.73* | *8.559* | *9.239* | *W= -2.735, P= .018* |
|  | Active | | | |  |
|  | *Pooled Mean Ranks*  *Negative* | *Pooled Mean Ranks*  *Positive* | *Averaged Median*  *Before Training* | *Averaged Median*  *After Training* |  |
| MMSE | *22.56* | *24.96* | *27.000* | *28.000* | *W= -4.120, P< .001* |
| RAVLT total | *24.36* | *33.47* | *38.903* | *45.507* | *W= -3.783, P= .005* |
| CVLT1 | *20.36* | *30.79* | *4.584* | *6.650* | *W= -3.978, P< .001* |
| CVLT total | *16.94* | *32.25* | *39.941* | *47.742* | *W= -4.561, P < .001* |
| CVLTD | *23.74* | *30.98* | *8.259* | *9.908* | *W= -3.605, P= .002* |
| Digit Span Backward | *21.30* | *26.96* | *4.004* | *4.488* | *W= -2.530, P= .032* |
|  | PTC | | | |  |
|  | *Pooled Mean Ranks*  *Negative* | *Pooled Mean Ranks*  *Positive* | *Averaged Median*  *Before Training* | *Averaged Median*  *After Training* |  |
| MoCA | *9.99* | *13.73* | *22.394* | *23.00* | *W= -2.039, P= .044* |
| RAVLT total | *12.43* | *17.51* | *37.546* | *44.117* | *W= -2.747, P= .021* |
| CVLT total | *11.56* | *16.41* | *41.187* | *48.359* | *W= -2.612, P= .019* |
| CVLTD | *12.32* | *16.08* | *8.175* | *10.869* | *W= -3.296, P= .021* |
| Digit Span Forward | *10.66* | *14.02* | *5.000* | *5.000* | *W= -2.331, P= .020* |
| Digit Span Backward | *9.12* | *12.16* | *4.000* | *4.000* | *W=-2.782, P= .006* |
| GDS | *13.46* | *7.58* | *2.000* | *0.000* | *W=* *-3.457, P=* *.001* |
| *Negative mean rank: score post<test score pre*  *Positive mean rank: test score post> test score pre* | | | | | |

Table 4: Among-groups comparisons results in non-ε4-carriers. Descriptive measures (pooled mean ranks of post-pre- scores) for each group (LLM, Active, PTC) are displayed.

| Neuropsychological tests | LLM | Active | PTC | Test results |
| --- | --- | --- | --- | --- |
| ***Cognitive Domain*** | | | | |
|  | *Pooled Mean Ranks* | *Pooled Mean Ranks* | *Pooled Mean Ranks* |  |
| MMSE | *57.11* | *70.00* | *54.77* | *χ^2^(2)= 4.631, P= .12* |
| MoCA | *60.75* | *64.68* | *56.39* | *χ^2^(2)=* *0.483, P=* *.80* |
| RAVLT1 | *61.13* | *62.48* | *62.73* | *χ^2^(2)=* *1.730, P=* *.49* |
| RAVLT total | *59.67* | *62.69* | *65.19* | *χ^2^(2)=* *1.745, P= .51* |
| RAVLTD | *62.93* | *61.79* | *60.60* | *χ^2^(2)=* *1.658,P= .54* |
| CVLT1 | *53.86* | *67.73* | *66.11* | *χ^2^(2)=* *5.291, P=0.12* |
| CVLT total | *56.74* | *67.69* | *60.43* | χ2(2)= 3.224, P= .28 |
| CVLTD | *57.78* | *61.58* | *71.33* | *χ^2^(2)=* *3.575, P= .26* |
| Trail B | *61.96* | *64.68* | *56.39* | *χ^2^(2)=* *1.261, P= .58* |
| Digit Span Forward | *58.62* | *61.85* | *69.07* | *χ^2^(2)=* *1.847, P= .44* |
| Digit Span Backward | *57.62* | *63.21* | *68.18* | *χ^2^(2)=* *1.845, P= .45* |
| ***Functionality*** | | | | |
| FUCAS | *55.59* | *65.65* | *67.07* | *χ^2^(2)= 3.296, P= .27* |
| FRSSD | *58.73* | *66.00* | *60.05* | *χ^2^(2)= 3.053, P= .35* |
| IADL | *59.63* | *65.26* | *59.80* | *χ^2^(2)= 2.054, P= .48* |
| ***Affective Domain*** | | | | |
| GDS | *69.64* | *61.65* | *47.46* | *χ^2^(2)= 6.604, P= .047^a^* |
| BAI | *60.18* | *64.09* | *56.13* | *χ^2^(2)= 1.887,P= .55* |
| BDI | *64.13* | *62.76* | *56.13* | *χ^2^(2)= 2.292,P= .46* |

*^a^ statistically significant value with P< .05*

Table 5: Among-groups comparisons results in ε4-carriers. Descriptive measures (pooled mean ranks of post-pre- scores) for each group (LLM, Active, PTC) are presented.

| Neuropsychological tests | LLM | Active | PTC | Test results |
| --- | --- | --- | --- | --- |
| ***Cognitive Domain*** | | | | |
|  | *Pooled Mean Ranks* | *Pooled Mean Ranks* | *Pooled Mean Ranks* |  |
| MMSE | *12.44* | *17.63* | *16.37* | *χ^2^(2)= 2.340, P= .31* |
| MoCA | *12.25* | *16.20* | *18.57* | *χ^2^(2)= 3.151, P= .25* |
| RAVLT1 | *14.99* | *15.52* | *14.36* | *χ^2^(2)= 1.053, P= .66* |
| RAVLT total | *14.57* | *14.94* | *15.88* | *χ^2^(2)= 1.790, P= .49* |
| RAVLTD | *14.48* | *15.96* | *14.74* | *χ^2^(2)= 1.545, P= .57* |
| CVLT1 | *14.09* | *14.67* | *17.12* | *χ^2^(2)= 1.631, P= .49* |
| CVLT total | *13.30* | *16.02* | *16.84* | *χ^2^(2)=* *1.703, P= .50* |
| CVLTD | *13.37* | *15.69* | *17.15* | *χ^2^(2)=* *1.860, P= .48* |
| Trail B | *12.16* | *15.52* | *19.61* | *χ^2^(2)=* *4.059, P= .18* |
| Digit Span Forward | *15.50* | *13.51* | *15.99* | *χ^2^(2)=* *1.624, P= .61* |
| Digit Span Backward | *14.42* | *14.68* | *16.49* | *χ^2^(2)=* *0.756, P= .72* |
| ***Functionality*** | | | | |
| FUCAS | *15.21* | *15.67* | *13.74* | *χ^2^(2)=* *1.176, P=* *.59* |
| FRSSD | *15.25* | *15.99* | *13.25* | *χ^2^(2)=* *1.977, P=* *.53* |
| IADL | *16.62* | *12.58* | *15.10* | *χ^2^(2)=* *2.860, P=* *.45* |
| ***Affective Domain*** | | | | |
| GDS | *16.87* | *17.29* | *8.57* | *χ^2^(2)= 5.653, P= .07* |
| BAI | *15.07* | *15.86* | *13.77* | *χ^2^(2)=* *1.771, P=* *.56* |
| BDI | *14.68* | *15.48* | *14.98* | *χ^2^(2)=* *1.040, P=* *.64* |

Table 6: Significant Score changes in the performance of ε4-carriers and non-ε4-carriers of the LLM, Active and PTC group at different cognitive tests.

| Neuropsychological tests | Group | | | | | | | | | | | | | Test results | |
| --- | --- | --- | --- | --- | --- | --- | --- | --- | --- | --- | --- | --- | --- | --- | --- |
| LLM – ε4-Carriers | | | | | | | | | | | | | | | |
|  | *Pooled Mean Ranks*  *Negative* | *Pooled Mean Ranks*  *Positive* | | | | *Averaged Median*  *Before Training* | | | | *Averaged Median*  *After Training* | | | |  | |
| Trail B | *7.03* | *6.87* | | | | *234.797* | | | | *185.516* | | | | *W= -2.179, P= .034* | |
| LLM – Non- ε4-carriers | | | | | | | | | | | | | | | |
| MMSE | *14.30* | | | | *20.36* | | | | *27.000* | | | | *28.000* | *W=-2.000, P= .047* | |
| MoCA | *20.24* | | | | *22.85* | | | | *22.915* | | | | *24.000* | *W= -2.228, P= .039* | |
| RAVLT1 | *17.33* | | | | *26.15* | | | | *4.523* | | | | *5.463* | *W = -2.992, P= .049* | |
| RAVLT total | *18.95* | | | | *26.06* | | | | *37.699* | | | | *43.220* | *W = -2.987, P= .036* | |
| CVLT1 | *14.47* | | | | *21.42* | | | | *4.751* | | | | *5.566* | *W= -2.554, P= .030* | |
| CVLT total | *18.88* | | | | *22.97* | | | | *41.605* | | | | *46.994* | *W = -3.410, P=.002* | |
| CVLTD | *14.49* | | | | *22.83* | | | | *8.4705* | | | | *9.598* | *W= -2.734, P= .023* | |
| Active - Non ε4-carriers | | | | | | | | | | | | | | | |
|  | *Pooled Mean Ranks*  *Negative* | | *Pooled Mean Ranks*  *Positive* | | | | *Averaged Median*  *Before Training* | | | | *Averaged Median*  *After Training* | | |  | |
| MMSE | *19.58* | | *21.34* | | | | *26.948* | | | | *28.000* | | | *W= -3.761, P< .001* | |
| MoCA | *17.28* | | *27.77* | | | | *22.997* | | | | *24.726* | | | *W= -2.392, P= .043* | |
| RAVLT total | *19.28* | | *28.41* | | | | *38.771* | | | | *45.806* | | | *W= -3.625, P= .013* | |
| CVLT1 | *14.54* | | *26.21* | | | | *4.507* | | | | *6.8315* | | | *W= -4.213, P< .001* | |
| CVLT total | *12.45* | | *26.75* | | | | *39.180* | | | | *48.408* | | | *W= -4.462, P< .001* | |
| CVLTD | *19.06* | | *25.64* | | | | *8.312* | | | | *10.002* | | | *W= -3.526, P= .002* | |
| Digit Span Backward | *17.41* | | *22.16* | | | | *4.000* | | | | *4.362* | | | *W= -2.281, P= .046* | |
| FUCAS | *17.66* | | *23.69* | | | | *43.798* | | | | *44.271* | | | *W=-3.230, P= .004* | |
| PTC - ε4-Carriers | | | | | | | | | | | | | | | |
|  | *Pooled Mean Ranks*  *Negative* | | | *Pooled Mean Ranks*  *Positive* | | | | *Averaged Median*  *Before Training* | | | | *Averaged Median*  *After Training* | | |  |
| Digit Span Backward | *2.50* | | | *3.75* | | | | *4.00* | | | | *5.00* | | | *W= -2.121, P= .034* |
| GDS | *3.50* | | | *0.00* | | | | *2.000* | | | | *0.000* | | | *W=-2.232, P= .026* |
| PTC – Non- ε4-carriers | | | | | | | | | | | | | | | |
| RAVLT total | *9.37* | | | *13.62* | | | | *37.509* | | | | | *44.118* | | *W=-2.593, P= .027* |
| CVLT total | *9.32* | | | *13.02* | | | | *41.230* | | | | | *47.803* | | *W=-2.368, P= .043* |
| CVLTD | *9.41* | | | *13.16* | | | | *7.871* | | | | | *10.822* | | *W=-2.992, P= .026* |
| Digit Span Forward | *9.02* | | | *10.96* | | | | *4.405* | | | | | *5.000* | | *W=-2.518, P= .012* |
| Digit Span Backward | *6.52* | | | *9.96* | | | | *4.000* | | | | | *4.000* | | *W= -2.219, P= .029* |
| FUCAS | *8.54* | | | *14.55* | | | | *43.537* | | | | | *44.505* | | *W= -2.299, P= .050* |
| GDS | *10.68* | | | *5.28* | | | | *2.000* | | | | | *0.000* | | *W= -2.831, P= .006* |
| *Negative mean rank: score post<test score pre*  *Positive mean rank: test score post> test score pre* | | | | | | | | | | | | | | | |
