# Supplementary material for: Web-Based Intervention Effects on Mild Cognitive Impairment Based on Apolipoprotein E Genotype: Quasi-Experimental Study
Source: J Med Internet Res. 2020 May 7;22(5):e14617. doi: 10.2196/14617 (PMC7243129; doi:10.2196/14617)

Multimedia Appendix 3

1.
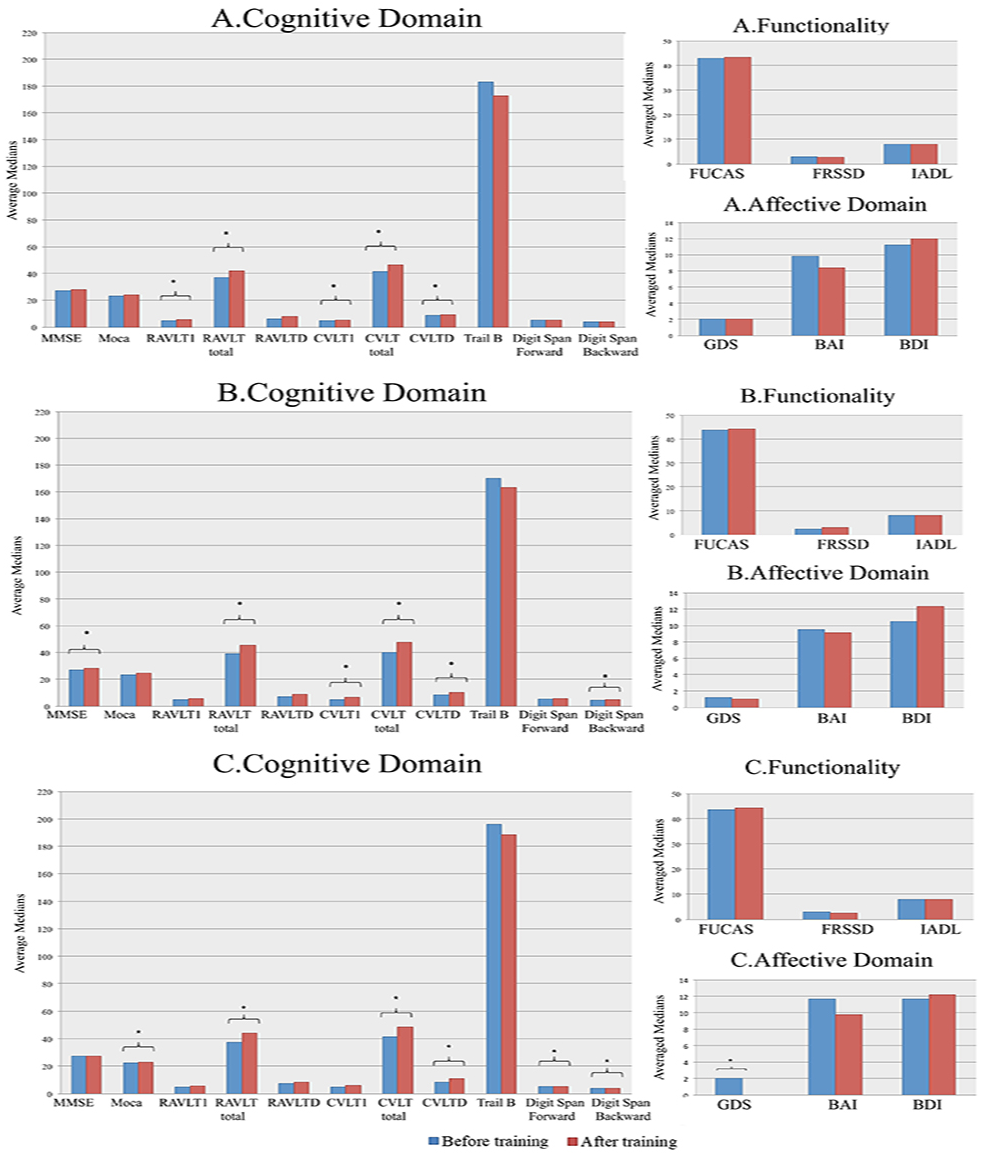
Long-Lasting Memories (Α), Active Control (Β), and Physical Training Control (C) groups (first, second, and third rows, respectively) before (blue) and after (red) training neuropsychological assessment. Statistically significant differences are indicated by * when *P*<.05.
2.
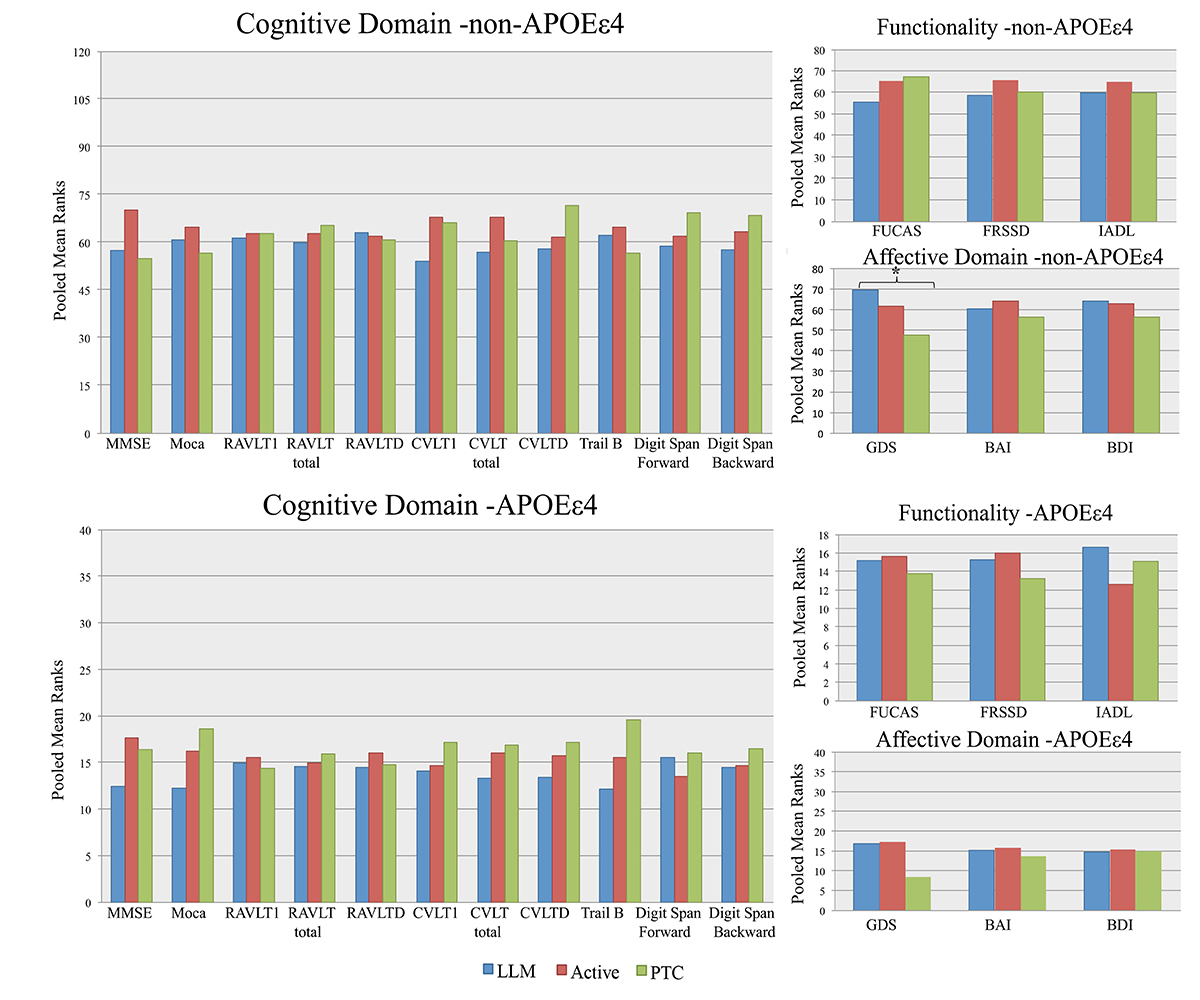
Among-group analysis comparing the scores’ differences of APOE non-ε4 carriers (first row) and APOE ε4 carriers (second row) at the two time points for Long-Lasting Memories (blue), Active Control (red), and Physical Training Control (green) groups. Statistically significant differences are indicated by * when *P*<.05.
3.
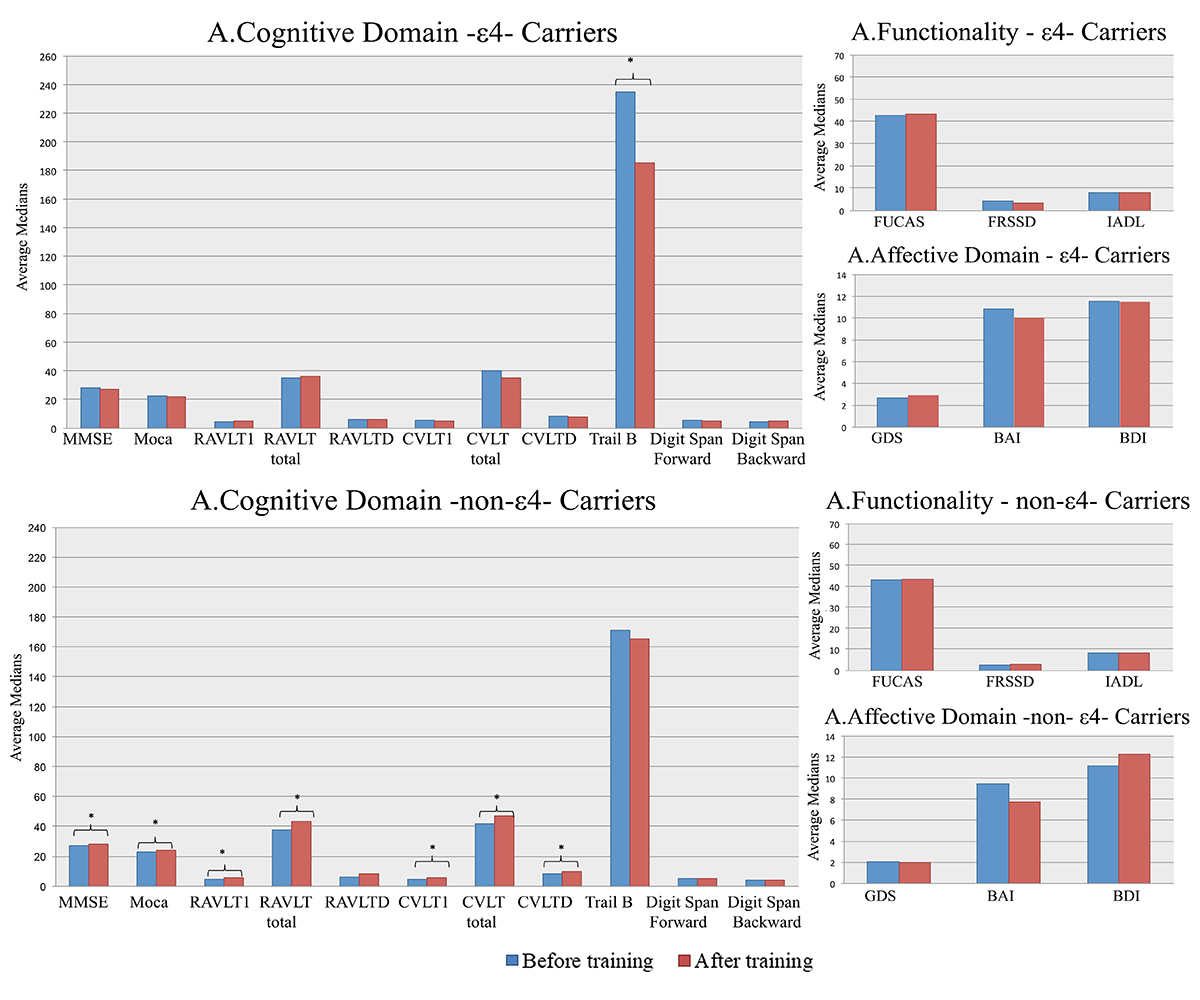
Long-Lasting Memories group APOE ε4 carriers/APOE non-ε4 carriers before (blue) and after (red) training neuropsychological assessment. Statistically significant differences are indicated by * when *P*<.05.
4. Active Control group APOE ε4 carriers/APOE non-ε4 carriers before (blue) and after (red) training neuropsychological assessment. Statistically significant differences are indicated by * when *P*<.05.


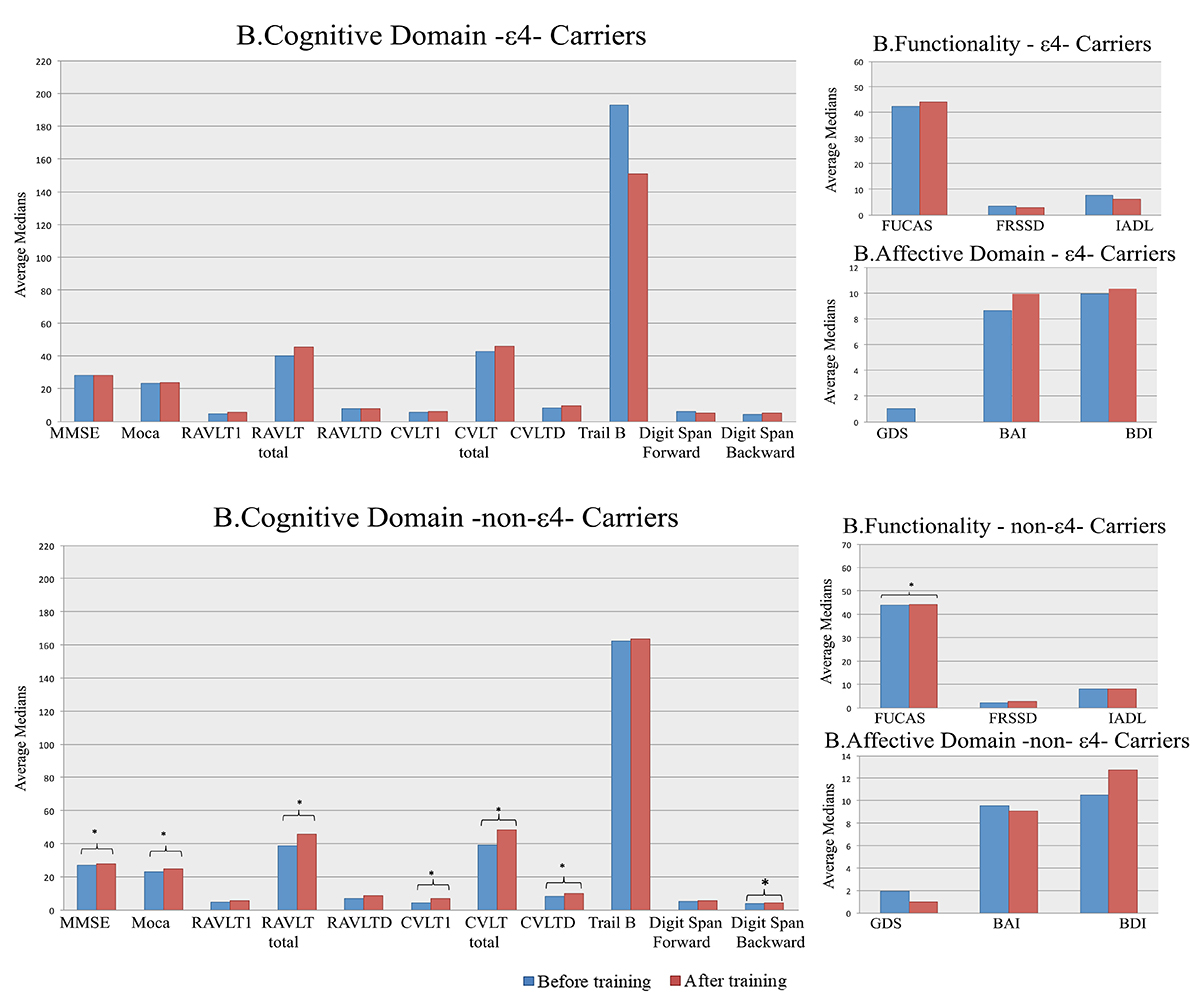


1. Physical Training Control group APOE ε4 carriers/APOE non-ε4 carriers before (blue) and after (red) training neuropsychological assessment. Statistically significant differences are indicated by * when *P*<.05.


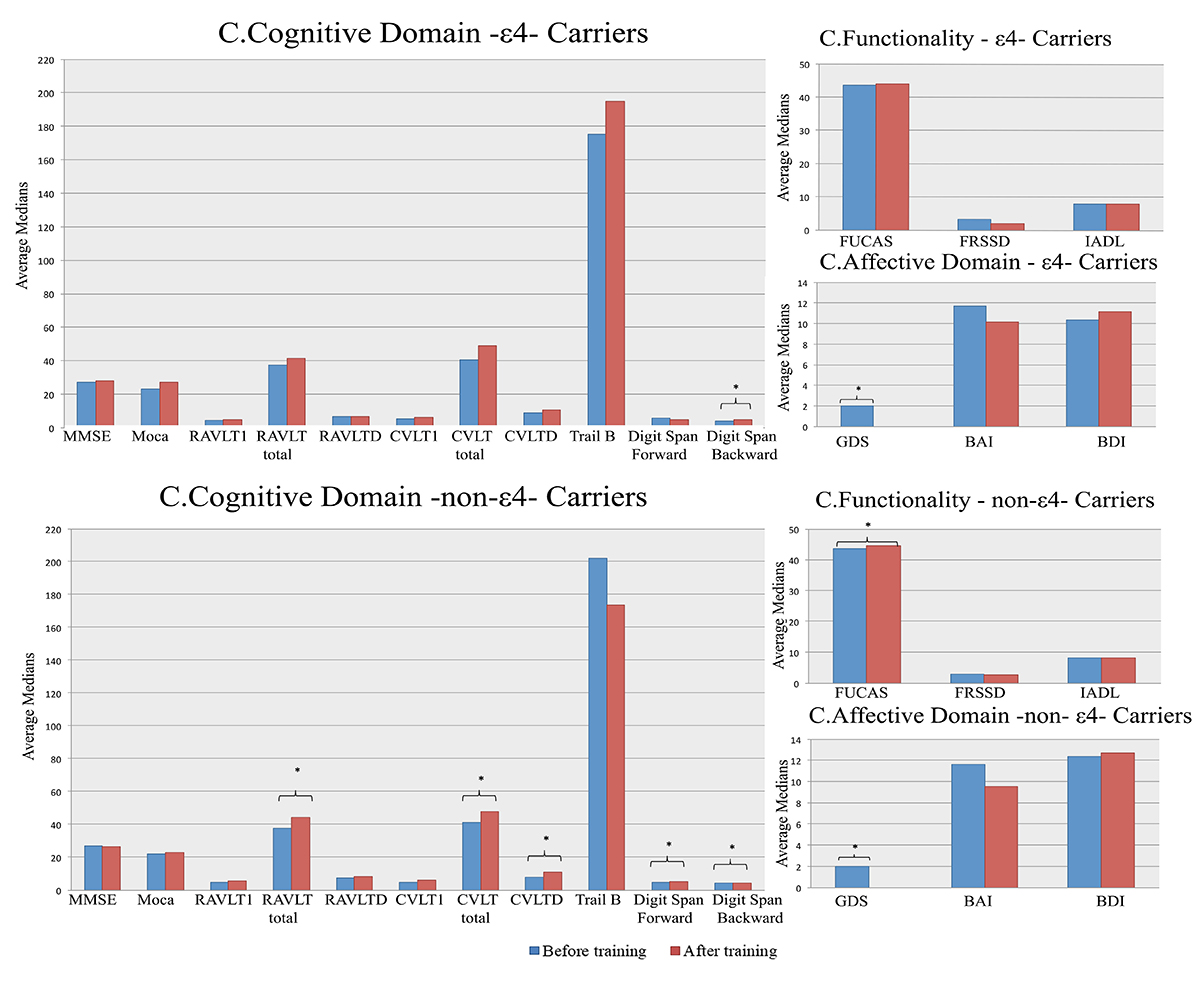

Supplement: Multimedia Appendix 3 [file jmir_v22i5e14617_app3.docx]
